# Assessing the level of evidence in the orthopaedic literature, 2013–2018: a review of 3449 articles in leading orthopaedic journals

**DOI:** 10.1186/s13037-020-00246-6

**Published:** 2020-05-16

**Authors:** Kitchai Luksameearunothai, Yash Chaudhry, Sorawut Thamyongkit, Xiaofeng Jia, Erik A. Hasenboehler

**Affiliations:** 1 Department of Orthopaedic Surgery, Johns Hopkins Bayview Medical Center, 4940 Eastern Ave, Baltimore, 21224 MD USA; 2 Department of Orthopaedic Surgery, Faculty of Medicine Vajira Hospital, Navamindradhiraj University, Bangkok, Thailand; 3 The Johns Hopkins Bloomberg School of Public Health, Baltimore, MD USA; 4 Department of Orthopaedic Surgery Adult and Trauma Division, The Johns Hopkins University Hospital, Baltimore, 21287 MD USA

**Keywords:** Level of evidence, Nontrauma research, Trauma research

## Abstract

**Background:**

In biomedical research, level of evidence (LOE) indicates the quality of a study. Recent studies evaluating orthopaedic trauma literature between 1998 and 2013 have indicated that LOE in this field has improved. The objective of this study was to determine the validity of one such study by 1) comparing our results and how they relate to more recent years of publications; and 2) assessing how our findings may be used to estimate future changes.

**Methods:**

A total of 3449 articles published from 2013 to 2018 in *The Journal of Orthopaedic Trauma* (JOT); *Journal of Bone and Joint Surgery, American Volume* (JBJS-Am); and *Clinical Orthopaedics and Related Research* (CORR) were evaluated for their LOE. Articles published in JBJS-Am or CORR were classified as trauma or nontrauma studies; articles published in JOT were considered trauma studies. Articles were assigned a LOE using guidance published by JBJS-Am in 2015.

**Results:**

The percentage of total high-level (level I or II) trauma and nontrauma articles published in JOT, JBJS-Am, and CORR decreased from 2013 to 2018 (trauma 23.1 to 19.2%, *p* = 0.190; nontrauma 28.8 to 24.9%, *p* = 0.037). JBJS-Am published the highest percentage of level-I trauma studies, and CORR published the lowest percentage of level-IV studies. JBJS-Am and CORR published higher percentages of level-I trauma studies and lower percentages of level-IV nontrauma studies than all trauma studies.

**Conclusions:**

Based on our results we cannot validate the findings of previous studies as we found the overall LOE of both trauma and nontrauma orthopaedic literature has decreased in recent years. JBJS-Am published a greater percentage of high-level studies than did JOT and CORR. Although the number and percentage of high-level studies published in JOT increased during the study period, it still lagged behind JBJS-Am and CORR.

## Background

Evidence-based medicine (EBM) is medical practice intended to optimize decision-making, treatment, and diagnosis based on well-designed research. The concept of level of evidence (LOE) was introduced in 1979 by the Canadian Task Force on the Periodic Health Examination [1] and was designed to support EBM, stratifying research designs according to their validity. The Oxford Centre for Evidence-Based Medicine (OCEBM) published the guidelines for LOE for prognostic, diagnostic, and therapeutic studies in 2009 [2]. Level-I research is the strongest evidence available, with data acquired through randomized controlled trials or meta-analysis of randomized controlled trials, whereas level-V evidence is the weakest evidence, representing cadaveric studies, case reports, or expert opinion.

Implementation of the LOE system encourages authors to improve the strength of their studies by avoiding bias, including control groups, and using methodical steps throughout the study [3]. The *Journal of Bone and Joint Surgery, American Volume* (JBJS-Am) was the first orthopaedic journal to apply the classification system and has assigned LOE for all studies it has published since 2003 [4].

In 2015, JBJS-Am updated their LOE criteria by modifying slightly the updated recommendations published by the OCEBM [5]. This change helped emphasize the clinical implications of research findings while implementing a more comprehensive assessment of the publication’s LOE. This update gave authors the flexibility to assign a grade of level I through IV, with a higher level indicating higher quality and a lower level indicating poorer quality or inconsiderable clinical effect.

Studies examining the LOE of scholarly orthopaedic articles have reported that journal impact factor is positively correlated with the proportion of “high-level” (level I or II) articles a journal publishes, and that journals with lower impact factors publish articles with an inconsistent LOE [6–9]. Prior studies such as the one conducted by Scheschuk et al. assessing LOE in orthopaedic literature between 1998 and 2013 have demonstrated a decrease in the number of low-level studies, an increase in high-level studies, and that orthopaedic trauma journals still publish a higher proportion of low-level articles [10–12]. The purpose of this study was to assess the validity of a previous published article [12] by 1) comparing our results and how they relate to more recent years of publications; and 2) assess how our findings may be used to estimate future changes.

## Methods

### Journal selection

Articles from the 3 most commonly used English-language journals publishing orthopaedic trauma and nontrauma research were evaluated for the LOE of their articles: the *Journal of Orthopaedic Trauma* (JOT) (trauma journal) and *Journal of Bone and Joint Surgery, American Volume* (JBJS-Am) and *Clinical Orthopaedics and Related Research* (CORR) (nontrauma journals). All articles published by these journals from 2013 through 2018 were evaluated. In accordance with the updated JBJS-Am LOE criteria [5] we defined high-level articles as level I or II and low-level articles as Level III or IV.

### Exclusion criteria

We excluded level-V studies (i.e., anatomical, animal, cadaveric, basic science, biomechanical, simulation, and educational studies; case reports; technical notes; expert opinions; surveys; publication analyses; and review articles) [13]. We also excluded studies with unclear methods (LOE not listed or study methods not clearly defined), those for which full text was unavailable, and those published in supplementary issues of the journals.

### Article categorization

#### Trauma vs. nontrauma

We categorized the articles in JBJS-Am and CORR as either trauma or nontrauma studies. Nontrauma studies were those concerning congenital or developmental abnormalities, arthroplasty, tumors, sports medicine, nontraumatic spine disorders, hand surgery, or nontraumatic foot and ankle surgery. Trauma studies were those concerning trauma to the upper or lower extremities, spine, pelvis, acetabulum, foot, or ankle.

### Study evaluation

Each study was reviewed by 1 of 2 authors. We assessed interobserver agreement by randomly selecting and assigning articles that met our inclusion criteria to reviewer 1 or reviewer 2, who assigned a LOE. In case of disagreement between reviewers, a third author (reviewer 3) was asked to judge the LOE. The percentage of agreement was 95% for study type (κ = 0.91) and 82% for LOE (κ = 0.73).

### Statistical analysis

The proportions of articles in each LOE were compared using chi-squared tests. The Cochran-Mantel-Haenszel test was used to adjust research years as a stratification factor. *P* < 0.05 was considered statistically significant.

**Fig. 1:**
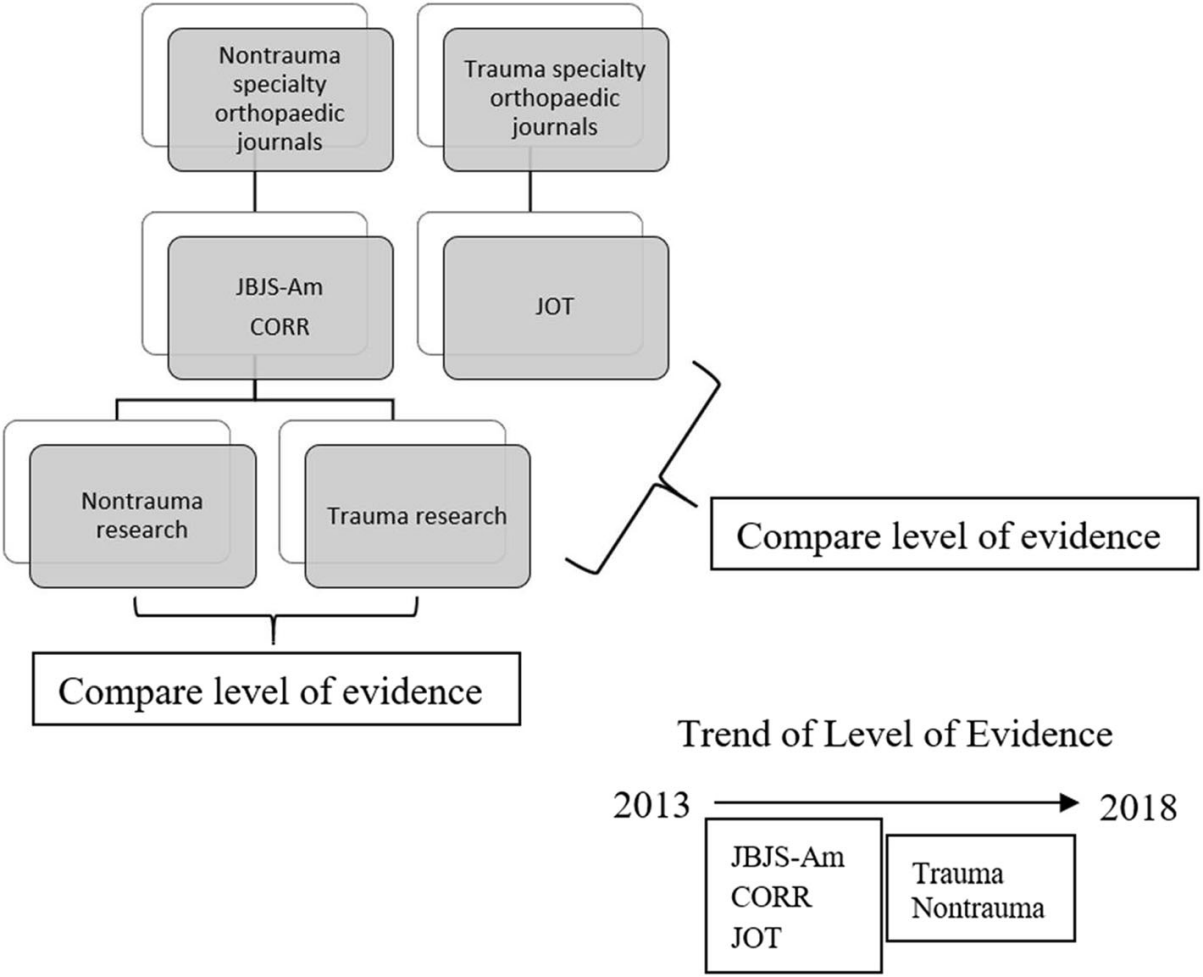
Study algorithm

After classifying the LOE of each article, we analyzed the following: 1) LOE of all orthopaedic trauma articles compared with all nontrauma articles; 2) LOE of JOT articles versus all trauma and nontrauma articles in JBJS-Am and CORR; 3) LOE of JOT articles versus JBJS-Am and CORR trauma articles only; 4) changes in LOE during the study period for JOT and for trauma articles in JBJS-Am and CORR by journal (Fig. 1); and 5) changes in LOE for all trauma and non-trauma articles over the duration of the study period. R, version 3.5.1 (R Foundation for Statistical Computing, Vienna, Austria), was used for statistical analysis.

## Results

During the study period, 4664 articles were published by the 3 journals, of which 3449 (74%) met our study criteria. The sample comprised 1378 articles (40%) from CORR, 1272 (37%) from JBJS-Am, and 799 (23%) from JOT. Only 1217 articles (35%) were classified as trauma studies across all 3 journals.

### LOE of all reviewed studies for all journals

**Fig. 2:**
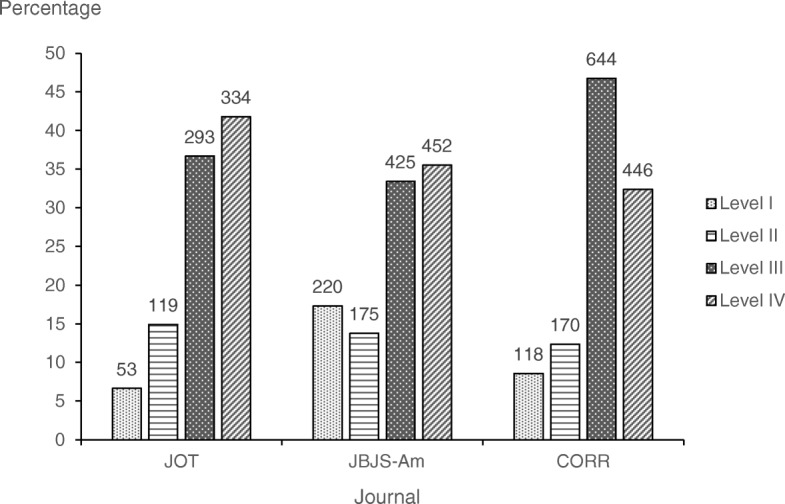
Numbers (above bars) and percentages of articles published, by level of evidence, from 2013 through 2018 in the *Journal of Orthopaedic Trauma* (JOT), *Clinical Orthopaedics and Related Research* (CORR), and the *Journal of Bone and Joint Surgery, American Volume* (JBJS-Am)

The most common LOE was level III (1362 articles, 39%), followed by level IV (1232 articles, 36%), level II (464 articles, 13%) and level I (391 articles, 11%) (Fig. 2). JBJS-Am published the highest percentage of level-I studies among the 3 journals (*P* < 0.001). CORR published the lowest percentage of level-IV studies (*P* < 0.001; Fig. 2).

### LOE of trauma vs. nontrauma studies

**Fig. 3:**
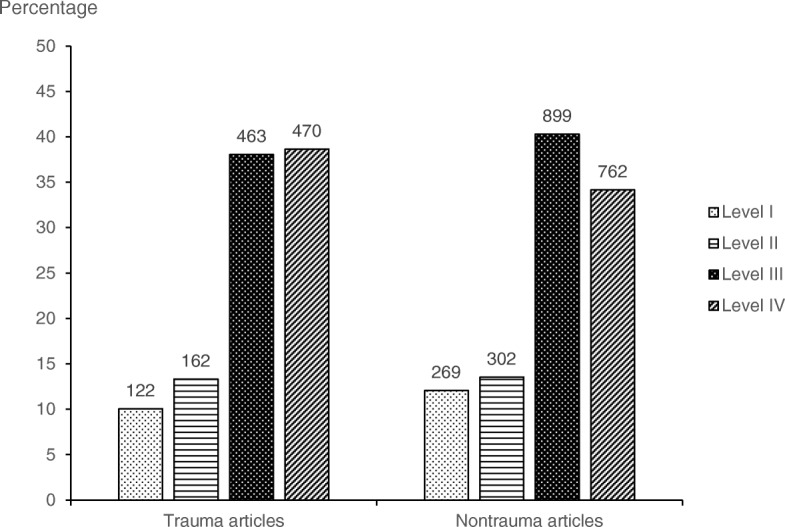
Numbers (above bars) and percentages of articles, by level of evidence and by orthopaedic trauma (*n* = 1217) versus orthopaedic nontrauma (*n* = 2232) topic, published from 2013 to 2018 in the *Journal of Orthopaedic Trauma*, *Clinical Orthopaedics and Related Research*, and the *Journal of Bone and Joint Surgery, American Volume*

Trauma literature (470 articles; 38.6%) had a significantly higher percentage of level IV articles than nontrauma (762 articles; 34.1%) (*P* = 0.01). The percentage of level I articles in nontrauma literature (269 articles; 12.1%) was higher than that of the trauma literature (122 articles; 10.0%), although this finding was not significant (*P* = 0.08) (Fig. 3). The total percentage of high-level articles (level I and II) over the study period for trauma and nontrauma literature was 25.6% (571) and 23.3% (284), respectively.

### LOE of trauma vs. nontrauma studies in JBJS-am and CORR

**Fig. 4:**
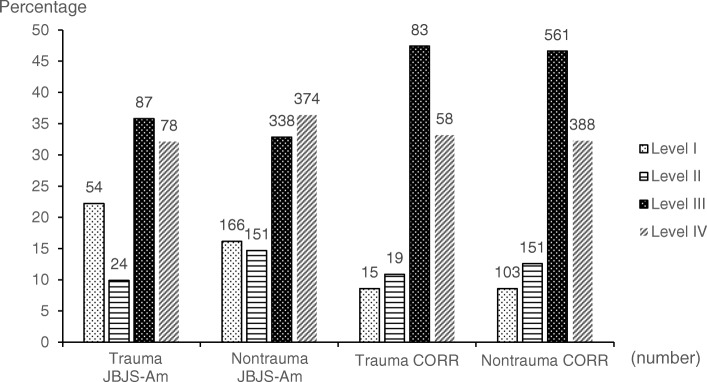
Numbers (above bars) and percentages of articles by level of evidence and by orthopaedic trauma versus orthopaedic nontrauma topic, published from 2013 to 2018 in *Clinical Orthopaedics and Related Research* (CORR) and the *Journal of Bone and Joint Surgery, American Volume* (JBJS-Am)

In JBJS-Am, we found a significant difference in the LOE between trauma and nontrauma studies (*P* = 0.029), with trauma (54 articles; 22.2%) demonstrating a higher percentage of level-I articles than nontrauma (166 articles; 16.1%). We found no significant difference in LOE between trauma and nontrauma articles in CORR (*P* = 0.937) (Fig. 4).

### High-level vs low-level studies by journal

**Fig. 5:**
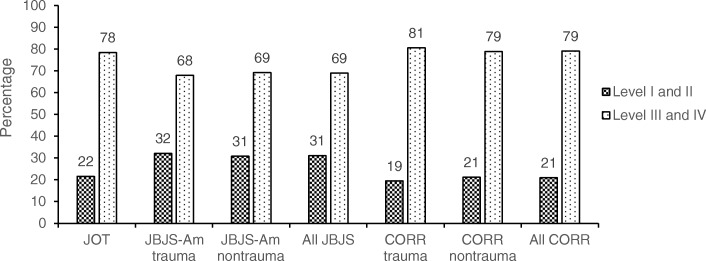
Percentages of high-level (level of evidence I or II) versus low-level (level of evidence III or IV) articles published from 2013 to 2018 in the *Journal of Orthopaedic Trauma* (JOT), *Clinical Orthopaedics and Related Research* (CORR), and the *Journal of Bone and Joint Surgery, American Volume* (JBJS-Am)

A significantly higher percentage of level-I and level-II studies were published in JBJS-Am (31%) compared with JOT (22%) and CORR (21%) (*P* < 0.001). There was no such difference when comparing JOT and CORR (*P* = 0.77; Fig. 5).

When analyzing the percentages of high-level studies by year, we found significant differences between JBJS-Am vs. JOT (*P* = 0.005) and JBJS-Am vs. CORR (*P* < 0.001). No such difference was found between JOT vs. CORR (*P* = 0.674). JOT published a smaller percentage of high-level studies (22%) compared with the percentage of high-level trauma studies published in JBJS-Am (32%) (*P* = 0.001). No difference was found between the percentages of high-level trauma studies published in JOT (22%) and CORR (19%) (*P* = 0.609). We also found no significant difference in LOE between trauma vs. nontrauma studies published by JBJS-Am (*P* = 0.695) or CORR (*P* = 0.608).

**Fig. 6:**
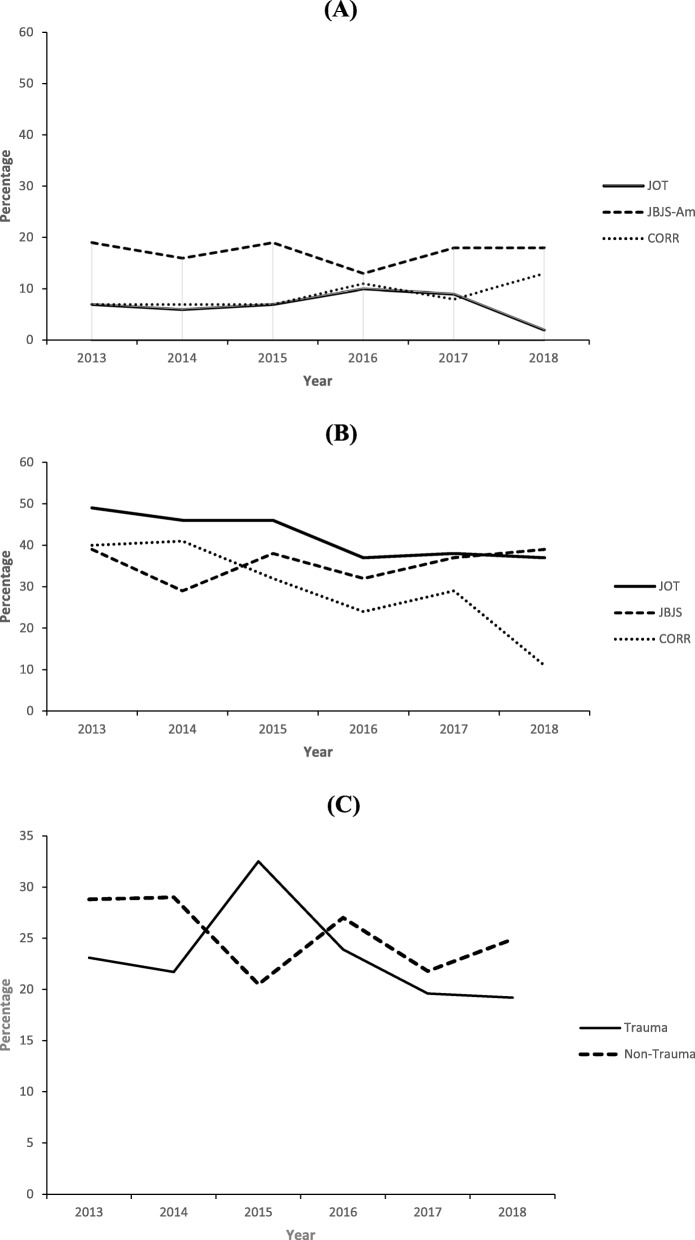
Changes in the percentages of (**a**) level-I trauma, (**b**) level-IV trauma, and (**c**) high-level (level-I or level-II) trauma and non-trauma articles published from 2013 to 2018 in the *Journal of Orthopaedic Trauma* (JOT), *Clinical Orthopaedics and Related Research* (CORR), and the *Journal of Bone and Joint Surgery, American Volume* (JBJS-Am)

When analyzing LOE in each journal over time, JOT had a significant decrease in level-I (7.3% in 2013 to 1.9% in 2018) and level-IV articles (49% in 2013 to 37% in 2018) and an increase in level-III articles (34% in 2013 to 45% in 2018). JBJS-Am had a significant decrease in level-II articles (21% in 2013 to 6.6% in 2018), and minimal changes in level-I, level-III, and level-IV articles. The LOE in CORR improved, with a significant decrease in level-IV articles (40% in 2013 to 11% in 2018), a significant increase in level-III articles (37% in 2013 to 62% in 2018), but minimal changes in level-I and level-II articles (Fig. 6a-b).

### High-level vs. low level studies over time (trauma vs non-trauma)

After combining all studies from the three included journals, we found that between 2013 and 2018, there was a non-significant downward trend in the percentage of high-level trauma articles (23% in 2013 to 19% in 2018; *P* = 0.190) and a significant downward trend in percentage of high-level non-trauma articles over time (29% in 2013 to 25% in 2018; *P* = 0.037) (Fig. 6c).

## Discussion

The purpose of this study was to assess the validity of the findings of the study conducted by Scheschuk et al. [12] by evaluating the LOE of trauma and nontrauma research published in 3 major orthopaedic journals, comparing it to their findings and examining it has changed in recent years, and assessing how our findings may be used to estimate future changes. From 2013 through 2018, we found that most articles published by these journals (63%) were low-level (level III or IV) studies, and most (63%) focused on treatment (as opposed to diagnosis, prognosis, or economics). Only 30.4% of trauma and 34.4% of non-trauma articles were classified as level I or level II, with downward trends for both (although only the non-trauma trend was significant). JBJS-Am published the highest percentages of level-I and level-II studies, and CORR published the highest percentage of level-III studies and the lowest percentage of level-IV studies. JOT published the lowest percentage of level-I studies of all 3 journals.

Our finding regarding the decrease in high-level studies deviates from previous literature assessing levels of evidence in orthopaedics over time. Scheschuk et al. examined JBJS-Am, CORR, and JOT articles for LOE in 1998, 2003, 2008, and 2013, reporting an upward trend in level-I and -II studies over their study period [12]. Cunningham et al. examined eight orthopaedic subspecialty journals for articles published in 2000, 2005, and 2010 and reported an increase the proportion of level-I and -II studies over time [10]. The current study provides an update and new look at the state of orthopaedic literature, both trauma and non-trauma. One potential explanation for the difference in studies is the increased use of national databases in orthopaedic research, particularly since 2013 [14, 15]. These databases can provide large volumes of retrospective data but are still considered level-III evidence. Another reason could be year-to-year variation; we saw a rise of high-level trauma articles and a decrease in high-level non-trauma articles in the year 2015. The selective sampling of certain years may have caused the prior studies to miss important data in the years between. Our findings suggest that the increased implementation of the LOE system has not been effective in improving the quality of orthopaedic literature in recent years; thus, we were unable to validate the findings of Scheschuk et al. [12].

Across all 3 journals, high-level trauma studies were published more often by JBJS-Am than by JOT or CORR. No significant differences in LOE were found between JBJS-Am and CORR when comparing all articles. Okike et al. showed that LOE was the only factor significantly associated with journals’ acceptance of submitted manuscripts, and that level-III and level-IV studies were least likely to be accepted by JBJS-Am [16]. Because of the nature of orthopaedic trauma, in which patients are often treated on an emergency basis, designing high-level studies, such as randomized controlled trials, is difficult. Level-III and level-IV studies are the most frequently published clinical orthopaedic trauma studies, likely because of their greater feasibility and lower cost compared with high-level studies. Blinding can be difficult to implement, and the use of placebo controls would be unethical in orthopaedic trauma studies. Further, obtaining a large enough sample size to achieve adequate statistical power in orthopaedic trauma is difficult. Bhandari et al. reported that the quality of randomized trials in JBJS-Am could be improved if blinding, randomization concealment, and patient inclusion/exclusion criteria were made consistent across studies [17]. Retrospective studies and case series, when performed with rigorous methods, were also considered valuable studies because of design methods.

Our study has several strengths. First, we analyzed all published articles during a 6-year period from 3 major orthopaedic journals that publish orthopaedic trauma research. This contrasts with previous publications, which evaluated articles in an inconsistent manner, reviewing only certain years across a time period and comparing them to articles that were published before the implementation of the LOE system established by JBJS-Am in 2003 [10–12]. Second, our comprehensive review eliminates selection bias that might arise from using an inconsistent review period. Third, we confirmed good interobserver agreement in LOE assessment across all 3 journals for the entire study period.

A limitation of our study is our use of the LOE grading system updated by JBJS-Am 2015. Although the system is simple and reproducible, it allows reviewers to grade LOE downward or upward based on the study quality and methodology used, which can be considered as a confounding bias to properly effect results. Furthermore, this may also create differences in LOE when graded by different reviewers.

## Conclusion

We found a decreasing proportion of high-level evidence across our study period, both in trauma and non-trauma articles. Of the 3 journals analyzed, JBJS-Am published the greatest percentage of high-level studies. JOT published a lower percentage of high-level articles than did JBJS-Am and CORR. A higher percentage of level-I articles were published in JBJS-Am and CORR, whereas no significant difference was found among the 3 journals.

## Data Availability

The datasets used and/or analyzed during the current study are available from the corresponding author on reasonable request.

## Abbreviations

EBM: Evidence-based medicine
LOE: level of evidence
OCEBM: Oxford Centre for Evidence-Based Medicine
JBJS-Am: Journal of Bone and Joint Surgery, American Volume
JOT: The Journal of Orthopaedic Trauma
CORR: Clinical Orthopaedics and Related Research
